# The Relationship between Preoperative Urine Culture and Post-Percutaneous Nephrostolithotomy Systemic Inflammatory Response Syndrome: A Single-Center Retrospective Study

**DOI:** 10.3390/jpm13020187

**Published:** 2023-01-20

**Authors:** Hongmin Zhou, Tiancheng Xie, Yuchen Gao, Xudong Yao, Yunfei Xu

**Affiliations:** Department of Urology, Shanghai Tenth People’s Hospital, Tongji University School of Medicine, Shanghai 200072, China

**Keywords:** preoperative urine culture, percutaneous nephrostolithotomy, systemic inflammatory response syndrome, retrospective study

## Abstract

Background: To predict the occurrence of systemic inflammatory response syndrome (SIRS) after percutaneous nephrostrolithotomy(PCNL), preoperative urine culture is a popular method, but the debate about its predictive value is ongoing. In order to better evaluate the value of urine culture before percutaneous nephrolithotomy, we conducted a single-center retrospective study. Methods: A total of 273 patients who received PCNL in Shanghai Tenth People’s Hospital from January 2018 to December 2020 were retrospectively evaluated. Urine culture results, bacterial profiles, and other clinical information were collected. The primary outcome observed was the occurrence of SIRS after PCNL. Univariate and multivariate logistic regression analysis was performed to determine the predictive factors of SIRS after PCNL. A nomogram was constructed using the predictive factors, and the receiver operating characteristic (ROC) curves and calibration plot were drawn. Results: Our results showed that there was a significant correlation between positive preoperative urine cultures and the occurrence of postoperative systemic inflammatory response syndrome. Meanwhile, diabetes, staghorn calculi, and operation time were also risk factors for postoperative systemic inflammatory response syndrome. Our results suggest that among the positive bacteria in urine culture before percutaneous nephrolithotomy, *Enterococcus faecalis* has become the dominant strain. Conclusion: Urine culture is still an important method of preoperative evaluation. A comprehensive evaluation of multiple risk factors should be undertaken and heeded to before percutaneous nephrostrolithotomy. In addition, the impact of changes in bacterial drug resistance is also worthy of attention.

## 1. Introduction

At present, percutaneous nephrostolithotomy (PCNL) is the first-line treatment for large renal (≥2 cm), cast, or staghorn calculi [1,2,3,4]. However, PCNL is also associated with complications [5,6], such as bleeding, colonic injury, and postoperative sepsis. Postoperative sepsis is a dangerous complication after PCNL [7], and can be life-threatening in severe cases.

Preoperative laboratory examination, including routine preoperative blood and urine tests, and urine culture can prevent postoperative systemic inflammatory response syndrome (SIRS) to a certain extent [8]. The percentage of leukocytes, neutrophils, and C-reactive protein (CRP) can reflect the severity of inflammation. Routine urine results can reflect the number of white blood cells (WBCs) in patients’ urine but cannot rule out the existence of non-inflammatory WBCs. Urine culture is the most used laboratory examination to assess preoperative urinary tract infection, infection-related flora, and drug sensitivity results.

Preoperative urine culture is significant in guiding antibiotic treatment and avoiding infectious complications after PCNL [9]. However, sometimes patients with a negative preoperative urine culture still have a risk of sepsis [10,11]. There are different opinions on the role of preoperative urine culture [12,13,14,15,16]. It has been reported that stone culture is a better predictor of postoperative sepsis than urine culture, but the only way to obtain stones is through surgery, not before surgery, greatly affecting the early predictive value of stone culture. Therefore, it is still important to evaluate the role of urine culture in the occurrence of sepsis after PCNL. It is worth noting that it usually takes several days to obtain the urine culture results. The delay in laboratory diagnosis of pathogenic pathogens often leads clinicians to start empirical antibiotic treatment, which may lead to the development of antimicrobial drug resistance [17]. Therefore, it is necessary to determine the bacterial profiles and characteristics of common urine bacteria in PCNL patients with positive urine culture as it will help with clinical diagnosis and treatment.

The purpose of this study was to observe the distribution characteristics of urinary bacteria in kidney stone patients with positive urine culture, to explore the predictive effect of preoperative urine culture on the occurrence of SIRS after surgery, and to establish a corresponding predictive model to help clinicians identify high-risk patients and conduct corresponding therapy in advance.

## 2. Materials and Methods

Our retrospective study was approved by the ethics committee at the Shanghai Tenth People’s hospital. We collected information on patients who underwent PCNL treatment and preoperative urine culture in the Department of Urology at Shanghai Tenth People’s Hospital between 2018 and 2020. Exclusion criteria included: (1) patients with tumors, blood system and immune system diseases, or cases of hyperthyroidism; (2) patients younger than 18 years old; (3) patients who had incomplete medical records. For patients with positive urine culture, appropriate antibiotics should have been used for at least 7 days according to the culture antibiogram results and urine cultures should have been repeated to ensure negative urine culture before PCNL. Patients with negative urine culture were given 1.5 g cefuroxime or 0.5 g levofloxacin intravenously 30 min before surgery for prevention. We recorded patients’ heart rate, blood pressure, body temperature, respiratory rate, and preoperative and postoperative laboratory examination results, operation time and other associated parameters, and any patient diseases. After PCNL, SIRS was defined when patients exhibited two of the following criteria: heart rate > 100 beats/min, WBC count ≥ 12 × 10^9^ cells/L or ≤4 × 10^9^ cells/L, body temperature higher than 38 °C or lower than 36 °C, or a respiratory rate higher than 20 breaths/min.

We analyzed the clinical data, preoperative and postoperative results between the negative urine culture (UC-) group and positive urine culture (UC+) group, and post-PCNL SIRS positive and negative groups, as well as the independent risk factors of preoperative positive urine culture and post-PCNL SIRS. All data were analyzed by SPSS (version 23.0) and several charts were made in Excel 2019. The nomogram was based on R. A chi-squared test or Fisher’s exact test was used to analyze categorical variables. The logistic regression model was established by forward linear stepwise regression. Continuous variables were tested for normality, and a Mann–Whitney U-test analysis was performed on those not conforming to a normal distribution. The *p*-value was of a two-tailed distribution. A *p* value < 0.05 indicates statistical significance.

## 3. Results

Table 1 lists the basic demographic data of patients in the UC- group and UC+ group. From 273 patients who underwent PCNL, 47 UC+ cases (17.2%) and 226 UC- cases (82.8%) were recognized. The mean age of the UC+ group was 56 (range: 47–64) and that of the UC- group was 60 (range:49–68). There was no significant difference between the two groups (*p* = 0.214). There were 188 male patients and 85 female patients. The female group (21 UC+ of 85 cases, 24.7%) had a significantly higher rate of urine culture positivity than the male group (26 UC+ of 188 cases, 13.8%) (*p* = 0.028). There were 21 staghorn calculi patients in the UC+ group and 49 in the UC- group (*p* = 0.001). The mean stone diameter in the UC+ group was 2.5 cm (range: 2.0–3.5 cm), and in the UC- group it was 2.0 cm (range: 1.5–2.5 cm) (*p* = 0.002). There were 32 patients (68.1%) whose urine was WBC ≥ 100 in the UC+ group, and 43 patients (19.0%) whose urine was WBC ≥ 100 in the UC- group (*p* < 0.001). There were 27 patients (57.4%) whose leukocyte esterase was ≥3 in the UC+ group, and 17 patients (7.5%) in the UC- group (*p* < 0.001). There were 10 urine nitrite positive patients (21.3%) in the UC+ group, and five (2.2%) in the UC- group (*p* < 0.001). After surgery, there were 18 patients in the UC+ group and 32 patients in the UC- group (*p* < 0.001) who developed SIRS. There were no significant differences between the two groups in the following factors: body mass index (*p* = 0.240), diabetes (*p* = 0.091), hypertension (*p* = 0.645), body temperature (*p* = 0.125), number of stones (*p* = 0.205), hydronephrosis (*p* = 0.556), preoperative blood neutrophils (*p* = 0.549), preoperative blood lymphocytes (*p* = 0.559), preoperative blood neutrophile-lymphocyte ratio (NLR) (*p* = 0.922), elevated CRP (*p* = 0.059), albumin-globulin ratio <1.5 (*p* = 0.725), serum creatinine (*p* = 0.190), and operation time (*p* = 0.877) (Table 1).

Table 2 lists the basic demographics of patients in the non-SIRS and SIRS groups. From 273 patients who underwent PCNL, 50 cases (18.3%) developed SIRS. The mean age of the non-SIRS group was 57 (range: 47–65), and that of the SIRS group was 55 (range: 48–62) (*p* = 0.443). There was no significant difference between the female group (18 SIRS cases of 85 cases, 21.2%) and male group (32 SIRS cases of 188 cases, 17%) (*p* = 0.411). There were 24 staghorn calculi patients in the SIRS group and 46 in the non-SIRS group (*p* < 0.001). The mean stone diameter in the SIRS group was 2.5 cm (range: 2.0–3.5 cm), and in the non-SIRS group it was 2.0 cm (range: 1.5–2.5 cm) (*p* < 0.001). There were 21 patients (42.0%) with urine WBC ≥100 in the SIRS group, and 54 patients (24.2%) in the non-SIRS group (*p* = 0.011). There were 15 patients (30.0%) whose leukocyte esterase was ≥3 in the SIRS group, and 29 patients (13.0%) in the non-SIRS group (*p* = 0.003). There were seven urine nitrite positive patients (14.0%) in the SIRS group, and eight urine nitrite positive patients (3.6%) in the non-SIRS group (*p* = 0.009). The mean preoperative lymphocyte count (cells −10^9^/L) in the SIRS group was 2.0 (range: 1.6–2.7), and that in the non-SIRS was 1.8 (range: 1.5–2.2) (*p* = 0.021). The mean preoperative NLR in the SIRS group was 2.0 (range: 1.4–2.5), and that in the non-SIRS was 2.2 (range: 1.8–3.0) (*p* = 0.022). The mean operation time of the SIRS group (78 min; range: 64–90 min) was significantly longer than in the non-SIRS group (64 min; range: 53–84) (*p* = 0.007). There were 18 UC+ patients (36%) in the SIRS group, which was significantly larger than the 29 UC+ patients in the non-SIRS group (13%) (*p* < 0.001) (Table 2).

Univariate analyses of the factors associated with post-PCNL SIRS are shown in Table 3. SIRS was more likely to occur in patients with staghorn calculi (*p* < 0.001), a large stone size (*p* < 0.001), preoperative urine WBC ≥ 100 (*p* = 0.012), preoperative leukocyte esterase ≥3+ (*p* = 0.004), positive urine nitrite (*p* = 0.007), longer operation time (*p* = 0.015), and UC+ (*p* < 0.001). We then conducted multivariate binary logistic regression analysis. Diabetes (odds ratio [OR] 2.666, 95% confidence interval [CI] 1.193–5.954, *p* = 0.017), staghorn calculi (OR 2.924, 95% CI 1.467–5.830, *p* = 0.002), operation time (OR 1.013, 95% CI 1.001–1.025, *p* = 0.036), and UC+ (OR 3.607, 95% CI 1.680–7.741, *p* = 0.001) were associated with the occurrence of post-PCNL SIRS (Table 3).

**Figure 1:**
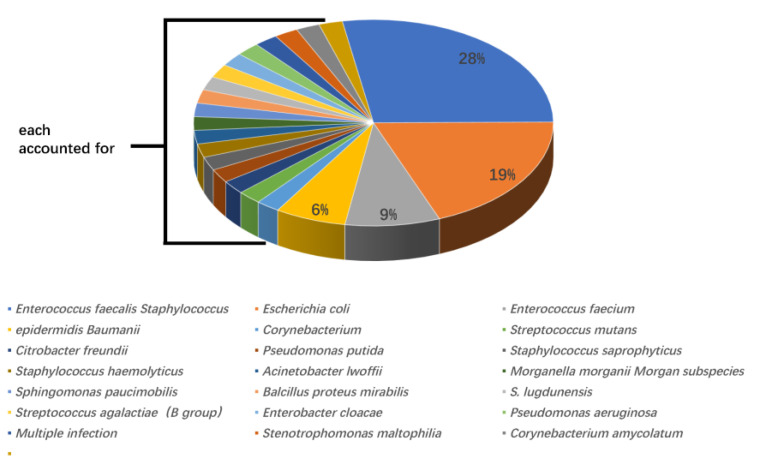
The genera and proportions of bacterial strains from patients with positive urine cultures.

**Figure 2:**
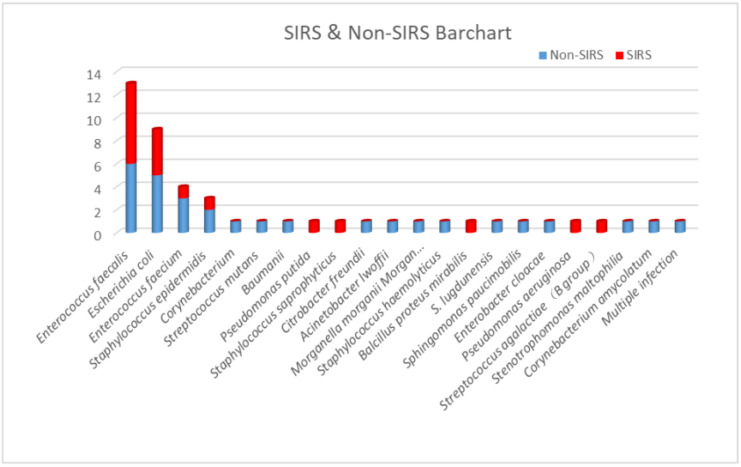
The proportion of post-PCNL SIRS in patients with different bacterial strains in urine cultures.

According to urine culture results, *Enterococcus faecalis* was the most widely recognized microorganism (13 cases, 28%), followed by *Escherichia coli* (9 cases, 19%), *Enterococcus faecium* (4 cases, 9%), and *Staphylococcus epidermidis* (3 cases, 6%). All other positive bacteria were only present in one case. Out of 13 cases, seven (53.8%) with *Enterococcus faecalis* developed post-PCNL SIRS, and four of nine cases (44.4%) with *Escherichia coli* developed post-PCNL SIRS. The proportions of SIRS caused by other UC+ bacteria were 25% for *Enterococcus faecium* (one in four cases) and 33.3% for *Staphylococcus epidermidis* (one in three cases) (Figure 1 and Figure 2).

**Figure 3:**
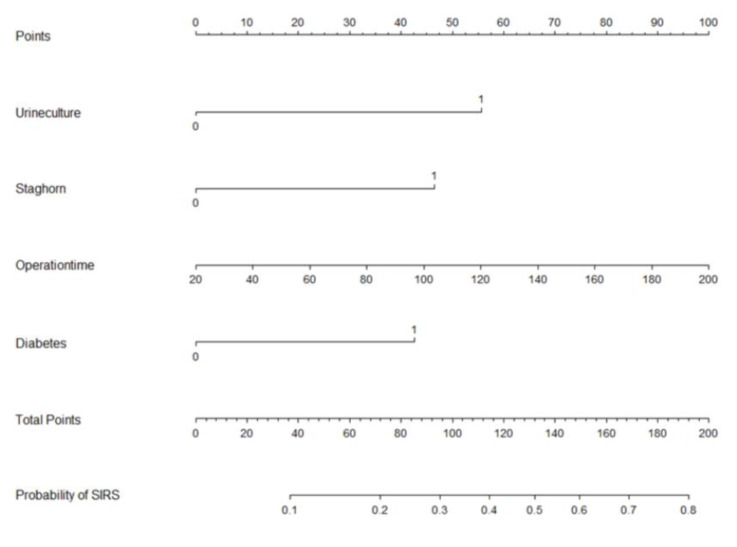
Nomogram with positive urine culture, staghorn calculi, operation time, and diabetes predicts the probability of post-PCNL SIRS.

**Figure 4:**
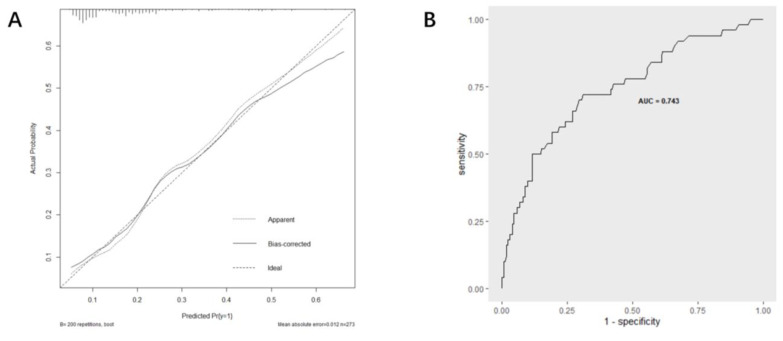
Evaluation of the predictive performance using calibration curve (**A**) and ROC curve (**B**).

Based on multi-factor analysis, a nomogram prediction model was established to calculate the cumulative probability of SIRS after PCNL (Figure 3). The incidence of SIRS can be calculated by adding together the points assigned to the four factors to obtain the total scale score. The calibration curve shows that the model fits well through the Hosmer–Lemeshow test, with a *p* value = 0.7148 (>0.05) indicating that there is no statistical difference between the approximate and ideal models (Figure 4A). At the same time, the area under our receiver operating characteristic curve is 0.743, which is very close to the C index (0.742), indicating that our model has good prediction value (Figure 4B).

## 4. Discussion

The main purpose of our study was to explore the predictive effect of preoperative urine culture results on the occurrence of postoperative SIRS, and on this basis, build a nomogram to predict the occurrence of postoperative SIRS after PCNL. Four independent risk factors—positive urine culture, staghorn calculi, operation time, and diabetes—were identified by multivariate logistic regression analysis to construct the nomogram. The AUC value of the nomogram was 0.743, indicating good predictive accuracy. In addition, we monitored the distribution of bacterial profiles in the urine of patients with kidney stones and found that *Enterococcus faecalis* was the main pathogen in urine.

The basic approach of PCNL is to establish a channel from the skin to the kidney at the waist. Through this channel, the nephroscope is inserted into the kidney, and the kidney stones are broken down and removed by laser, ultrasound, and other lithotripsy tools. PCNL is a modern, minimally invasive technique for the treatment of renal calculi [1,2,3], which has almost eliminated open surgery. Compared with open surgery, PCNL has the advantages of less injury, less pain, complete stone removal, and rapid recovery. Compared with laparoscopic lithotomy, PCNL has little effect on the structure of the kidney and surrounding areas and does not affect renal surgery in the future. Compared with extracorporeal lithotripsy, PCNL has a shorter treatment cycle, an immediate effect, and reduced impact on renal function.

However, we should note that complications of PCNL include SIRS, intraoperative bleeding, postoperative bleeding, perforation and tear of renal collecting system, water electrolyte disorder, peripheral organ injury, urinary cyst formation, wound nonunion, and more [4,5,6]. The occurrence of postoperative SIRS may seriously endanger the patient’s life and health, due to septic shock.

Recent studies have shown that preoperative inflammation, operation time, and stone size may increase the incidence of SIRS after PCNL [18,19]. Inflammatory indexing in blood and urine is often used as a marker to judge the level of inflammation and the probability of postoperative SIRS. Urine culture, as a simple method, is widely used in hospitals as a bacterial test before PCNL [20,21]. Usually, greater care is taken with UC+ patients, including necessary measures such as antibiotics to reduce the incidence of postoperative SIRS. However, in some UC-patients, SIRS still occurs after the operation [9,10]. Therefore, there are different views on the reliability of preoperative urine culture results [22].

Some hospitals have carried out stone culture detection, and relevant studies have shown that stone culture is more accurate than urine culture in predicting the occurrence of postoperative SIRS [15,16]. Stone culture can most directly reflect the internal flora of stones, but it can only be carried out during or after operation and cannot provide predictive results to help doctors preoperatively.

Our results showed that there was a significant correlation between preoperative urine culture results and postoperative SIRS. Meanwhile, the occurrence of SIRS after the operation was related to the following factors: diabetes mellitus, staghorn calculi, and operation time. We should also take note of the maximum diameter of stones, preoperative urinary WBC (≥100), preoperative leukocyte esterase (≥3+), and positive urinary nitrite. Therefore, we believe that preoperative urine culture is still important evidence to judge the occurrence of postoperative SIRS. We should be cautious and take preventive measures in these cases.

The main pathogen found in the urine of patients with kidney stones is Escherichia coli. According to the results of urine culture of patients with kidney stones, Chen et al. found that 54% of pathogens were *Escherichia coli*, followed by *Enterococcus* (9.4%) and *Pseudomonas mirabilis* (7.6%) [22,23]. In a study from Romania, *Escherichia coli* (35.98%) was still the most common urinary pathogen, but the proportion of *Enterococcus* reached 19.73% [24]. In our center’s research, *Enterococcus faecalis* surpassed *Escherichia coli* and became the dominant strain among the positive bacteria in urine culture. This result may be different from common *Escherichia coli*. It is important to note that although urinary tract infections are mainly caused by Gram-negative bacteria, Gram-positive bacteria have also become important pathogens, and are commonly associated with elderly patients, diabetes, pregnancy, and indwelling catheters [25,26]. We speculate that the change of the bacterial spectrum may be related to factors such as bacterial drug resistance and small sample size, which need further study.

Several limitations should be pointed out. Firstly, the clinical data came from a single-center institution, which may lead to potential selection bias. Secondly, as this was a retrospective analysis, and the complete SOFA scoring parameters were not available in our institution’s database, we could only perform as comprehensive an analysis as possible based on the available data. Although the SIRS concept is no longer included in the Third International Consensus Definitions for Sepsis and Septic Shock, the parameters used to define SIRS are easily measurable and reproducible in everyday urological clinical practice compared to SOFA assessment parameters. Therefore, the SIRS criteria may remain a useful screening tool for identifying patients with sepsis. Finally, the sample size was small; we plan to include more patients for analysis in future studies.

## 5. Conclusions

Preoperative urine culture is still an important method to predict post-PCNL SIRS. We should undertake and take heed of a preoperative comprehensive evaluation of multiple risk factors, including diabetes mellitus, staghorn calculi, and operation time. Based on the preoperative urine culture results and other risk factors, we developed a nomogram that may be important for early identification of SIRS after PCNL. At the same time, the changes of urinary bacteria profiles in patients with kidney stones are also worthy of attention.

## Figures and Tables

**Table 1:** Clinical and preoperative factors between UC (−) and UC (+) groups.

Variables	UC (−)	UC (+)	*p*
Age (years)	56 (47–64)	60 (49–68)	0.214
Gender			**0.028**
Male, *n* (%)	162 (86.2%)	26 (13.8%)	
Female, *n* (%)	64 (75.3%)	21 (24.7%)	
BMI (Kg/m^2^)	24.6 (22.5–27.0)	23.4 (22.0–27.2)	0.240
Diabetes, *n* (%)	44 (19.5%)	4 (8.5%)	0.091
Hypertension, *n* (%)	88 (38.9%)	20 (42.6%)	0.645
Body temperature >37.5 °C	11 (4.9%)	5 (10.6%)	0.125
Number of stones			0.205
Solitary, *n* (%)	47 (20.8%)	6 (12.8%)	
Multiple, *n* (%)	179 (79.2%)	41 (87.2%)	
Staghorn calculi, *n* (%)	49 (21.7%)	21 (44.7%)	**0.001**
Hydronephrosis, *n* (%)	195 (86.3%)	39 (83.0%)	0.556
Largest stone size (cm)	2.0 (1.5–2.5)	2.5 (2.0–3.5)	**0.002**
Urine WBC ≥ 100, *n* (%)	43 (19.0%)	32 (68.1%)	**<0.001**
Leukocyte esterase 3+ or higher, n (%)	17 (7.5%)	27 (57.4%)	**<0.001**
Positive urine nitrite, *n* (%)	5 (2.2%)	10 (21.3%)	**<0.001**
Preoperative neutrophile (10^9^/L)	4.0 (3.3–5.0)	4.1 (3.2–5.5)	0.549
Preoperative lymphocyte (10^9^/L)	1.8 (1.5–2.2)	2.0 (1.4–2.4)	0.559
Preoperative neutrophile-lymphocyte ratio	2.2 (1.7–2.9)	2.2 (1.6–3.1)	0.922
Elevated CRP, *n* (%)	48 (21.2%)	16 (34.0%)	0.059
AGR < 1.5, *n* (%)	155 (68.6%)	31 (66.0%)	0.725
Serum creatinine			0.190
Normal, *n* (%)	170 (75.2%)	31 (66.0%)	
Abnormal, *n* (%)	56 (24.8%)	16 (34.0%)	
Operation time (min)	70 (53–85)	65 (53–86)	0.877
SIRS, *n* (%)	32 (14.2%)	18 (38.3%)	**<0.001**

BMI, body mass index; WBC, white blood cell; NLR, neutrophile-lymphocyte ratio; CRP, C-reactive protein; AGR, albumin-globulin ratio. Bold values indicate statistical significance (*p* < 0.05).

**Table 2:** Clinical and preoperative factors between SIRS and non-SIRS groups.

Variables	Non-SIRS	SIRS	*p*
Age (years)	57 (47–65)	55 (48–62)	0.443
Gender			0.411
Male, *n* (%)	156 (70.0%)	32 (64.0%)	
Female, *n* (%)	67 (30.0%)	18 (36.0%)	
BMI (Kg/m^2^)	24.5 (22.2–27.2)	22.9 (22.6–25.8)	0.509
Diabetes, *n* (%)	35 (15.7%)	13 (26.0%)	0.084
Hypertension, *n* (%)	87 (39.0%)	21 (42.0%)	0.696
Body temperature >37.5 °C	13 (5.8%)	3 (6.0%)	0.963
Number of stones			0.284
Solitary, *n* (%)	46 (20.6%)	7 (14.0%)	
Multiple, *n* (%)	177 (79.4%)	43 (86.0%)	
Staghorn calculi, *n* (%)	46 (20.6%)	24 (48.0%)	**<0.001**
Hydronephrosis, *n* (%)	191 (85.7%)	43 (86.0%)	0.949
Largest stone size (cm)	2.0 (1.5–2.5)	2.5 (2.0–3.5)	**<0.001**
Urine WBC ≥ 100, *n* (%)	54 (24.2%)	21 (42.0%)	**0.011**
Leukocyte esterase 3+ or higher, *n* (%)	29 (13.0%)	15 (30.0%)	**0.003**
Positive urine nitrite, *n* (%)	8 (3.6%)	7 (14.0%)	**0.009**
Preoperative neutrophile (10^9^/L)	4.1 (3.3–5.1)	4.0 (3.2–5.0)	0.484
Preoperative lymphocyte (10^9^/L)	1.8 (1.5–2.2)	2.0 (1.6–2.7)	**0.021**
Preoperative NLR	2.2 (1.8–3.0)	2.0 (1.4–2.5)	**0.022**
Elevated CRP, *n* (%)	52 (23.3%)	12 (24.0%)	0.918
AGR < 1.5, *n* (%)	151 (67.7%)	35 (70.0%)	0.754
Serum creatinine			0.437
Normal, *n* (%)	162 (72.6%)	39 (78.0%)	
Abnormal, *n* (%)	61 (27.4%)	11 (22.0%)	
Operation time (min)	64 (53–84)	78 (64–90)	**0.007**
Positive urine culture, *n* (%)	29 (13.0%)	18 (36.0%)	**<0.001**

BMI, body mass index; WBC, white blood cell; NLR, neutrophile-lymphocyte ratio; CRP, C-reactive protein; AGR, albumin-globulin ratio. Bold values indicate statistical significance (*p* < 0.05).

**Table 3:** Univariate analysis and multivariate binary logistic regression analysis.

Variables	Univariate Analysis			Multivariate Analysis		
	OR	95% CI	*p*	OR	95% CI	*p*
Age	0.992	0.967–1.017	0.519			
Gender	0.764	0.401–1.455	0.412			
Male/female						
BMI	0.970	0.893–1.054	0.477			
Diabetes	1.887	0.912–3.907	**0.087**	2.666	1.193–5.954	**0.017**
Hypertension	1.132	0.607–2.110	0.696			
Body temperature >37.5 °C	1.031	0.283–3.763	0.963			
Number of stones	1.596	0.674–3.781	0.288			
Multiple/solitary						
Staghorn calculi	3.552	1.868–6.754	**<0.001**	2.924	1.467–5.830	**0.002**
Hydronephrosis	1.029	0.426–2.487	0.949			
Largest stone size	1.925	1.360–2.723	**<0.001**			
Urine WBC ≥ 100	2.266	1.195–4.297	**0.012**			
Leukocyte esterase 3+ or higher	2.867	1.396–5.889	**0.004**			
Positive urine nitrite	4.375	1.507–12.703	**0.007**			
Preoperative neutrophile	1.056	0.967–1.153	0.224			
Preoperative lymphocyte	1.060	0.905–1.243	0.470			
Preoperative NLR	0.966	0.837–1.116	0.639			
Elevated CRP	1.018	0.506–2.132	0.918			
AGR < 1.5	1.113	0.571–2.167	0.754			
Serum creatinine	0.749	0.361–1.556	0.439			
Abnormal/normal						
Operation time (min)	1.014	1.003–1.025	**0.015**	1.013	1.001–1.025	**0.036**
Positive urine culture	3.763	1.874–7.554	**<0.001**	3.607	1.680–7.741	**0.001**

BMI, body mass index; WBC, white blood cell; NLR, neutrophile-lymphocyte ratio; CRP, C-reactive protein; AGR, albumin-globulin ratio. Bold values indicate statistical significance (*p* < 0.05).

## Data Availability

The datasets that support the findings of this study are included within the article, including figures and tables. Any other data used to support the findings of this study are available from the corresponding author on request.
